# Factors Associated with Maintaining the Mental Health of Employees after the Fukushima Nuclear Disaster: Findings from Companies Located in the Evacuation Area

**DOI:** 10.3390/ijerph15010053

**Published:** 2017-12-31

**Authors:** Masatsugu Orui, Yuriko Suzuki, Aya Goto, Seiji Yasumura

**Affiliations:** 1 Department of Public Health, Fukushima Medical University School of Medicine, Fukushima 960-1295, Japan; yrsuzuki@ncnp.go.jp (Y.S.); agoto@fmu.ac.jp (A.G.); yasumura@fmu.ac.jp (S.Y.); 2 Department of Adult Mental Health, National Center of Neurology and Psychiatry, National Institute of Mental Health, Tokyo 187-8553, Japan; 3 Center for Integrated Science and Humanities & International Community health, Fukushima Medical University School of Medicine, Fukushima 960-1295, Japan

**Keywords:** occupational health, mental health, nuclear disaster, epidemiology, health management

## Abstract

After the nuclear disaster in Fukushima on 11 March 2011, some businesses were permitted to continue operating even though they were located in the evacuation area designated by the Japanese government. The aim of this study was to examine differences in the mental health status, workplace, living environment, and lifestyle of employees in the evacuation and non-evacuation areas. We also investigated factors related to their mental health status. Data for this cross-sectional study were collected from the questionnaire responses of 647 employees at three medium-sized manufacturing companies in the evacuation and non-evacuation areas. Through a cross-tabulation analysis, employees who worked at companies in the evacuation areas showed an increase in the duration of overtime work, work burden, and commute time, and had experienced separation from family members due to the radiation disaster and perceived radiation risks. The results of a multivariate logistic regression analysis showed that, even in a harsh workplace and living environment, being younger, participating regularly in physical activity, having a social network (Lubben Social Network Scale-6 ≤ 12), laughing frequently, and feeling satisfied with one’s workplace and domestic life were significantly associated with maintaining a healthy mental health status after the disaster. These findings are applicable for workers’ health management measures after disasters.

## 1. Introduction

The Great East Japan Earthquake that occurred on 11 March 2011, generated a massive tsunami, and caused enormous damage to the Pacific Coast of Japan. Subsequently, the tsunami hit the Fukushima Daiichi Nuclear Power Plant operated by the Tokyo Electric Power Company. This accident caused radiation disasters in the Fukushima Prefecture and necessitated the long-term evacuation of residents from many surrounding municipalities. Due to the nuclear disaster, the Japanese government designated evacuation areas according to spatial radiation dose rates. The evacuation areas were classified into three categories: (1) difficult-to-return areas, with a radiation dose rate ≥50 millisieverts (mSv) per year; (2) residence restriction areas, with a radiation dose rate greater than or equal to 20 and less than 50 mSv per year; and (3) areas where evacuation orders are ready to be lifted, with a radiation dose rate of less than 20 mSv per year. Residents of these areas were forced to relocate to non-evacuation regions and were not allowed to stay overnight after the disaster. However, evacuees and employees who worked at companies in the residence restriction areas and the areas where evacuation orders were ready to be lifted were permitted to temporarily enter. Therefore, companies located in these areas were able to continue operating [1]. 

Devastating natural disasters and their aftermath cause psychological distress in affected individuals. In Fukushima, the earthquake, tsunami, and nuclear disaster led to a mandatory evacuation of people from the surrounding region. Consequently, residents were forced to relocate to non-evacuation areas and live in stressful situations, separated from family members, after losing housing, and having to adjust to new circumstances [2,3,4,5]. Moreover, a previous study that investigated the disaster- and work-related stressors and mental health status of public servants found that not taking a non-work day each week or working more than 100 h of overtime per month led to an increased risk of mental distress [6,7]. Therefore, employees of companies in the evacuation areas might have a heavy workload and face increased risk of psychological distress because of the stressors in their personal lives and workplaces. 

Conversely, previous studies have reported that several factors affect maintaining mental health, including regular leisure activities such as hobbies, exercise, or sports; sufficient sleep; having a social network; laughing daily; and maintaining a work-life balance [8,9,10,11,12,13,14,15]. Owing to these factors, employees from evacuation areas may be able to maintain their mental health status despite the drastic changes in their domestic lives or workplaces.

Some employees reported increases in their work burden or overtime work, or changes in their domestic lifestyle after large-scale disasters [6,7,16]. Therefore, the present study aimed to examine (1) differences in the mental health status, the workplace, living environment, and lifestyle of employees in the evacuation and non-evacuation areas; and (2) factors related to maintaining the mental health status of employees in the evacuation area despite drastic changes in their workplaces and living environments. The findings will be useful for health promotion strategies for occupational health in the current post-disaster situation and in the aftermath of future disasters.

## 2. Materials and Methods 

### 2.1. Study Design

**Figure 1:**
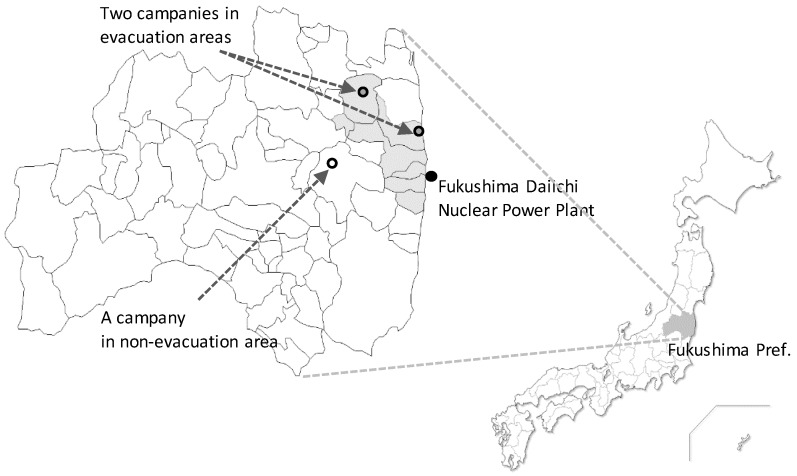
Location of the three companies in the evacuation and non-evacuation areas. Two medium-sized manufacturing companies in evacuation areas (Minami-Soma City and Iitate Village) and a medium-sized manufacturing company in a non-evacuation area (Tamura City) in the Fukushima Prefecture.

This study was based on cross-sectional data that we collected from a questionnaire survey distributed to employees at two medium-sized manufacturing companies (300 employees or less) in evacuation areas, and a medium-sized manufacturing company in a non-evacuation area (Figure 1). The distances from the Fukushima Daiichi Nuclear Power Plant to these companies in the evacuation area were 15 km and 40 km. The distance to the company in the non-evacuation area was 45 km. These companies appoint general health and safety managers to ensure employees are safe and are kept healthy in the workplace, which includes the prevention and treatment of diseases and injuries among employees, as regulated by the Industrial Safety and Health Act. Therefore, the companies sufficiently implemented health management measures, including annual health checks, before and after the nuclear disaster.

A questionnaire survey was distributed using a placement method for employees of the subject companies from September to November 2016. As this study targeted all employees in the three companies without exclusionary criteria, 383 responses were received for the evacuation areas and 264 for the non-evacuation area. The survey was approved by the ethical review committee of Fukushima Medical University on 29 July 2016 (No. 2797).

### 2.2. Measurements

We categorized the survey items as follows: (1) change in the workplace environment from the pre-disaster situation in terms of amount of overtime work, work burden, and commute time; (2) perception of radiation risks; (3) change in the living environment including relocation from an evacuation area, separation of family members due to the nuclear disaster; (4) change in lifestyle such as change in physical activity or sleeping time; (5) social network (Lubben Social Network Scale-6 (LSNS-6), Japanese version); (6) frequency of laughing; (6) satisfaction with current work status and domestic life; and (7) change and current subjective mental health status and current psychological distress (Kessler 6-item scale (K6)). The perceived risk of radiation exposure, LSNS-6, Japanese version, and the K6 item scale are validated measurements, and the others are investigator-designed queries.

#### 2.2.1. Change in the Workplace Environment 

Identifying changes in the workplace environment of employees in the evacuation areas was one of the primary aims of the investigation, to aid efforts in maintaining health and safety in the workplace. We investigated changes after the nuclear disaster, including employees’ amount of overtime work, work burden, and commute time, as the workplace environment variables, which were related to work stressors [6,7,17]. These factors were measured on a three-point scale: increase, no change, and decrease.

#### 2.2.2. Perception of Radiation Risks

Since the perception of radiation risks is a specific stressor after a nuclear disaster, we examined it as a stressor affecting employees’ mental health status. To evaluate the perception of radiation risks, the participants were asked questions such as, “What do you think the likelihood is of damage to your health (e.g., cancer onset) in later life as a result of your current level of radiation exposure?” [18]. These questions were answered using a four-point Likert scale as follows: ‘very unlikely’, ‘unlikely’, ‘likely’, or ‘very likely’. In the analysis, we categorized participants who had answered ‘very unlikely’ or ‘unlikely’ into the same group, which we called the low perceived risk group. Likewise, participants who had answered ‘likely’ or ‘very likely’ were classified into the high perceived risk group.

#### 2.2.3. Change in Living Environment

We investigated variables for change in the living environment such as relocation from an evacuation area and separation of family members due to the nuclear disaster. We defined participants as having experienced relocation from an evacuation area if their address as of 11 March 2011 was in Namie Town or Iitate Village, both places for which evacuation orders were issued for the total area of the municipality (as of August 2016). In addition, we considered participants as having experienced relocation from an evacuation area if they indicated that they currently lived in temporary housing or reconstructed public housing. 

As separation from family members due to the nuclear disaster could influence employees’ mental health status [19], it was used as a disaster-related experience variable assessed by the question, “Have you experienced living apart from your family who originally lived with you due to this nuclear disaster?”

#### 2.2.4. Change of Lifestyle

To evaluate lifestyle changes that might be related to employees’ mental health, we investigated changes in physical activity and sleeping time after the disaster [8,9]. These lifestyle factors were measured on a three-point scale: increase, no change, and decrease.

#### 2.2.5. Social Network

For social network variables, we used the LSNS-6, Japanese version [20], with the following six questions: (1) “How many relatives do you see or hear from at least once a month?”; (2) “How many relatives do you feel comfortable talking with about private matters?”; (3) “How many relatives do you feel close to such that you could call on them for help?”; (4) “How many of your friends do you see or hear from at least once a month?”; (5) “How many friends do you feel comfortable talking with about private matters?”; and (6) “How many friends do you feel close to such that you could call on them for help?” The participants answered these questions on a five-point scale (0 = none, 1 = one, 2 = two, 3 = three, or four, 4 = five to eight, 5 = nine or more). We classified respondents with 11 points or fewer as socially isolated [20].

#### 2.2.6. Frequency of Laughing

To assess the participants’ frequency of laughing, we used the standard single-item question, “How often do you laugh out loud?” The responses were: ‘never or almost never’, ‘1–3 times per month’, ‘1–5 times per week’, or ‘almost every day’ [13]. We divided the participants into two categories, ‘laughed almost every day’ and ‘laughed 1–5 times per week or less’ based on the previous study [21].

#### 2.2.7. Satisfaction with Current Workplace and Domestic Life 

Satisfaction with current workplace and domestic life were measured with the following items: “I am satisfied with my job” and “I am satisfied with my family life”. The participants responded on a four-point scale: ‘very satisfied’, ‘satisfied’, ‘unsatisfied’, and ‘very unsatisfied’. These items were taken from “The Brief Job Stress Questionnaire”, used to screen workers’ stress-related symptoms and status, which was introduced by the Ministry of Health, Labor, and Welfare in December 2015 [22].

#### 2.2.8. Changing and Current Subjective Mental Health Status and Current Psychological Distress

Changing subjective mental health status in comparison to before the disaster was measured on a three-point scale: improved, unchanged, and deteriorated. Current subjective mental health status was measured on a five-point scale: ‘very good’, ‘good’, ‘unremarkable’, ‘poor’, and ‘very poor’.

To assess psychological distress status, we used the K6. The K6 scale is used to screen for non-specific serious mental illnesses, including Diagnostic and Statistical Manual of Mental Disorders, Fifth Edition (DSM-IV) mood and anxiety disorders. The score range is from 0 to 24 points. Those scoring 0–12 points were classified as having probable mild–moderate/no psychological distress, and those scoring 13–24 points were classified as probably having serious psychological distress [23]. This study used the Japanese version of the K6, which has been empirically validated as an independent means of screening for mental distress among evacuees [24].

### 2.3. Stressors and Protective Factors for Employees’ Mental Health

We defined change in the workplace environment [6,7] and the perception of radiation risks as stressors on employees’ mental health status [25]. Also, maintaining one’s pre-disaster lifestyle, having a social network, laughing frequently, and feeling satisfied with one’s current work status and domestic life were considered protective factors for employees’ mental health [8,9,10,11,12,13,14,15]. Among the protective factors, maintaining physical activity, sleeping time, an adequate social network, and frequency of laughing were considered self-care behaviors that could maintain employees’ mental health status [8,9,10,11,12,13]. Satisfaction with current workplace and domestic life were considered employee care by managers [14,15].

### 2.4. Definition of Maintaining Employees’ Mental Health Status

Since we focused on factors related to maintaining employees’ mental health status after the disaster, we defined maintaining mental health status as follows. Participants were considered to be maintaining their mental health status if they (1) answered both ‘improved’ for change in the perception of subjective mental health status and ‘very good’ or ‘good’ to the question on current subjective mental health status; or (2) answered both ‘unchanged’ for change in the perception of subjective mental health status and ‘very good’, ‘good’, or ‘unremarkable’ to the question on current subjective mental health status.

### 2.5. Statistical Analysis

We performed a chi-square test and used multivariate logistic regression models to examine the differences in the workplace, living environment, and lifestyle of employees who worked in the evacuation and non-evacuation areas, and factors related to maintaining their mental health status. Statistical significance was evaluated using two-sided, design-based tests with a 5% level of significance. All statistical analyses were performed using SPSS 23.0 (IBM Corp., Armonk, NY, USA).

## 3. Results

### 3.1. Participants

**Figure 2:**
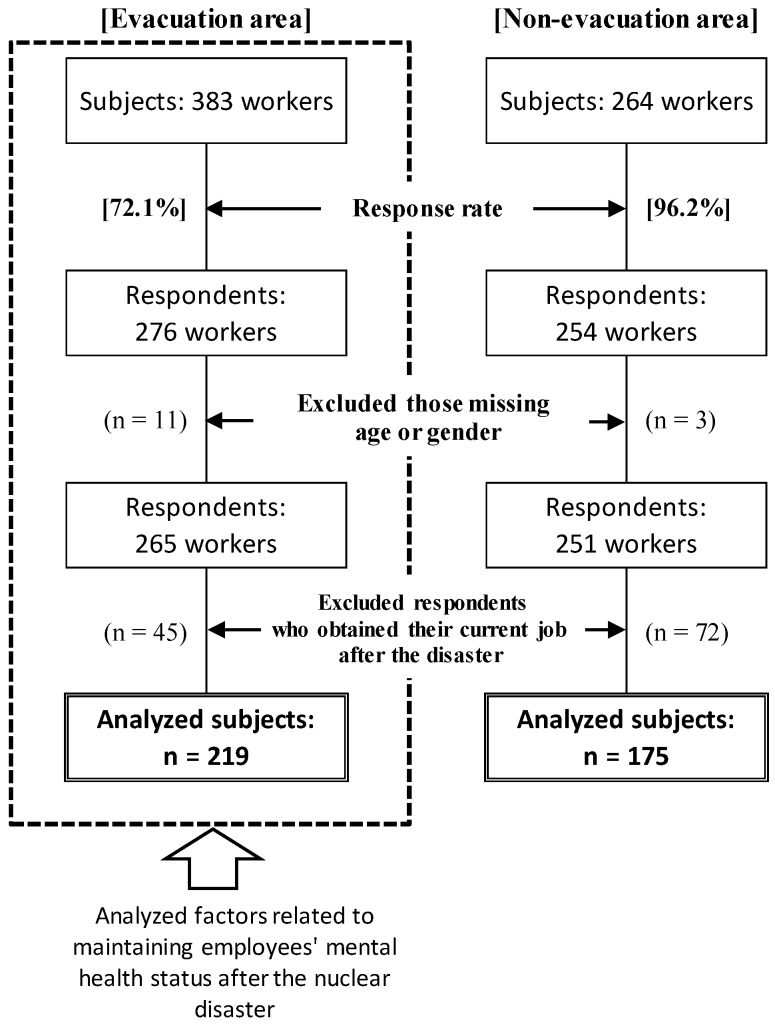
Sample selection from companies in the evacuation and non-evacuation areas.

Among the 647 subjects, 530 people responded to the questionnaire, for a response rate of 72.1% the evacuation areas and 96.2% in the non-evacuation area. Fourteen respondents who did not provide their age or gender information were excluded. Moreover, we excluded 117 respondents who had obtained their current job after the disaster from the chi-square test and multiple logistic regression models. Then, the data of 394 respondents (219 respondents in evacuation areas, 175 respondents in a non-evacuation area) were analyzed (Figure 2). In terms of gender, there were more male employees than female employees in each area, and the majority of all employees worked in production processes. Moreover, “relocation from an evacuation area” and “separation of family members due to the nuclear disaster” were significantly higher among employees who worked in evacuation areas. Since gender and age distribution were different between the subjects in the evacuation and non-evacuation area, we adjusted for age and gender in a multivariate logistic regression analysis (Table 1). 

Among the 647 subjects, 276 workers in the evacuation area and 254 workers in the non-evacuation area responded to the questionnaire. After excluding respondents who were missing age and gender information, and who obtained their current job after this disaster, we analyzed 219 subjects in the evacuation area and 175 subjects in the non-evacuation area.

### 3.2. Differences in the Workplace, Living Environment, and Lifestyle of Employees in the Evacuation and Non-Evacuation Areas

Table 2 shows the differences in status in the workplace, living environment, and lifestyle of employees in the evacuation and non-evacuation areas. Employees who worked in evacuation areas showed deteriorated statuses in the workplace environment in terms of amount of overtime work, work burden, and commute time, in comparison to employees who worked in the non-evacuation area. They had a significantly higher rate of perception of radiation risks and change in the living environment or workplace environment, including relocation from an evacuation area and separation of family members due to the nuclear disaster. Among protective factors for employees’ mental health status, employees in evacuation areas did not maintain their physical activity or sleep time in comparison to those in the non-evacuation area. Among employees in the evacuation area, the proportion of decreased physical activity or sleep time were higher than those who worked in the non-evacuation area. Moreover, employees who worked in evacuation areas felt significantly less satisfaction with their workplace when compared to their counterparts who worked in the non-evacuation area.

### 3.3. Prevalence of Maintaining Employees’ Mental Health Status in Evacuation Areas

K6 points increased as current subjective mental health status declined, which may be a reasonable indicator for current subjective mental health status. As for maintaining mental health status, 112 (51.9%) respondents were able to maintain their mental health status. We called these respondents the “group maintaining mental health status” (Table 3). 

### 3.4. Factors Related to Maintaining Employees’ Mental Health Status after the Nuclear Disaster in Evacuation Areas

In a cross-tabulation analysis, increased work burden and perception of radiation risks were higher among employees in evacuation areas who had a deteriorated status or unhealthy mental health status. Moreover, maintaining physical activity and sleep duration, having a strong social network, laughing frequently, and feeling satisfied with one’s work status and domestic life were significantly associated with maintaining mental health status (Table 4).

Table 5 shows the results of a multivariate logistic regression analysis for maintaining employees’ mental health status after the nuclear disaster. Model 1 included the variables of age, gender, and stressors on employees’ mental health for employees in evacuation areas who faced increased burdens in the workplace after the disaster (odds ratio (OR): 0.81, 95% confidence interval (CI): 0.68–0.96), perceived high risks of radiation exposure regarding delayed effects (OR: 0.81, 95% CI: 0.70–0.94), and could not significantly maintain their mental health status. Model 2 included protective factors for employees’ mental health status. Among employees in the evacuation area, age (OR: 0.96, 95% CI: 0.92–0.99), gender (OR: 3.55, 95% CI: 1.20–10.5), regular physical activity (OR: 1.31, 95% CI: 1.07–1.62), having a social network (OR: 1.23, 95% CI: 1.01–1.49), laughing frequently (OR: 1.29, 95% CI: 1.02–1.62), and satisfaction with one’s work status (OR: 1.38, 95% CI: 1.12–1.69) and domestic life (OR: 1.26, 95% CI: 1.01–1.57) were significantly associated with maintaining mental health status after the disaster. The significant negative association between increased burden, perceived high risk of radiation, and maintaining mental health status disappeared in Model 2, which included protective factors for employees’ mental health.

## 4. Discussion

### 4.1. Differences in the Workplace, Living Environment, and Lifestyle of Employees in the Evacuation and Non-Evacuation Areas

Our findings showed differences in the workplace, living environment, and lifestyle of employees who worked in evacuation areas compared to those who did not. Approximately half of the employees who worked in evacuation areas had to relocate outside of their original living places due to the evacuation; therefore, they had longer commutes than before [17]. Additionally, in a previous study of the workplace status of public servants in disaster-stricken areas after the Great East Japan Earthquake, 15.9% of workers suffered burnout even though more than three-quarters of the respondents were not involved in disaster-related work [26]. Additionally, the Ministry of Health, Labor, and Welfare reported that the percentage of effective job offers, which reflects the number of workers being sought by companies as regular or temporary staff, has consistently been increasing after the disaster [27]. These findings highlight the harsh workplace conditions that follow a severe disaster, as there is an increased demand for reconstruction business services. Subsequently, workers might experience greater work burdens. Among the lifestyle changes of employees in this study who worked in evacuation areas, regular physical activity decreased. This might be related to increased work burden, changes in the living environment due to replacements [28], or anxiety about radiation exposure [29]. Moreover, our findings implied that deteriorating mental health status among the employees (45.4% in evacuation areas, 16.2% in the non-evacuation area; Table 3) could lead to difficulties in maintaining sleep duration (33.2% in evacuation areas, 12.6% in the non-evacuation area) [30]. This was assumed to be due to the drastic changes in the workplace and living environment of employees in evacuation areas. Also, employees who worked in evacuation areas perceived radiation risks at a significant level, even though approximately half of them were non-evacuees. This might show that working within an evacuation area is linked to perceived radiation risks regardless of whether the employees were evacuees or not.

### 4.2. Factors Related to Maintaining Employees’ Mental Health Status after the Nuclear Disaster

Those employees who maintained their mental health status in evacuation areas accounted for 51.9% of the respondents (112/216 employees). The majority of the employees who worked in evacuation areas maintained their mental health status despite experiencing drastic changes in their workplaces and living environments. As for the variables for stressors on employees’ mental health status, deteriorating mental health status was significantly associated with increasing burden in the workplace in the Model 1 analysis. However, the significant association with work burden disappeared when the protective factors were added (physical activity, keeping sleeping time, having a social network, laughing frequently, and satisfaction with one’s workplace and domestic life) [8,9,10,11,12,13,14,15]. Therefore, mental health status could be maintained with protective factors, even when work burden increased.

Regarding protective factors for employees’ mental health status, a nationwide population-based study that followed participants for six years indicated that regular exercise or sports was significantly related to maintaining mental health status [8]. Furthermore, a previous study reported that laughter may lower the risk of subjective poor health [13]. Regular physical activities or laughing frequently might work protectively to maintain employees’ mental health status, although our investigation, designed as a cross-sectional study, did not demonstrate causality. In a previous study following the Great East Japan Earthquake, social networks were considered an important factor influencing mental health outcomes, and high social capital played an important role in protecting mental health [31]. Also, individuals in communities with high social capital suffered less from post-traumatic stress [10]. These findings support our finding; that is, high social capital led to employees’ maintaining their mental health status after a major disaster. Finally, satisfaction with the workplace and domestic life was most significantly associated with maintaining mental health status among the protective factors in our setting. A previous large-scale study in Switzerland of the employed population aged 20 to 64 found that workers’ work–life imbalance was a risk factor affecting mental health, and employees with self-reported work–life conflict presented a significantly higher relative risk of poor self-rated health, negative emotions, and depression [15]. Although this study did not directly measure work–life balance among employees, our findings show that those who felt satisfied with their workplace and domestic life had a well-balanced work and domestic life, and consequently, they could maintain their mental health status.

In summary, regular physical activity and laughing frequently serve as protective factors for employees’ mental health. Moreover, work–life balance also had positive effects on mental health status, even when employees were faced with drastic changes in their workplaces or domestic lives following massive disasters.

### 4.3. Limitations and Strengths

The present study has a few limitations. The first limitation is causality. Our findings were based on a cross-sectional study design. Therefore, we could not determine whether mental health status among employees could maintain their regular physical activities or sleep duration, having a social network, or frequency of laughing. Second, control selection bias might exist in the present study; the control group may not be truly representative of the non-evacuation area. Even employees in the non-evacuation area might have been affected by the nuclear disaster, because their company was located close to an evacuation area. Moreover, approximately 10% of respondents in the non-evacuation area experienced separation from family members. However, a previous study of psychological distress among 1709 Japanese employees showed that the K6 point greater than or equal to 13 was 10.8% [32], which was almost equivalent to or more than that of the employees in this study in the non-evacuation area (K6 point ≥ 13; 7.5%). Moreover, we obtained the data from only one company located in the non-evacuation area, which was imbalanced compared to the number of companies in the evacuation area. The reason for this was few companies cooperated as a control in this study. The third limitation is the difference in the response rate between the evacuation and non-evacuation employees. A previous study showed that mental health status might affect the response rate to a survey, suggesting that non-response was associated with poor mental health status [33]. There might be many employees in the evacuation area experiencing psychological distress who could not answer the survey, which might be underestimated in our findings. The forth limitation is recall bias. The respondents in evacuation areas could have been more likely to indicate that they had been affected by the nuclear disaster or changes in their work status or domestic life than the respondents in the non-evacuation area. The change in the workplace environment (e.g., work burden), change of lifestyle (e.g., change in physical activity or sleep duration), frequency of laughing, and satisfaction with current workplace and domestic life were subjective measurements. Therefore, it is necessary to be cautious when interpreting the findings. Fifth, multiple collinearities between maintaining mental health status and satisfaction with the workplace and domestic life might exist. Since both the dependent and independent variables were subjective, they may have been correlated with each other. However, in the Pearson’s correlation analysis, since the correlation coefficient was less than 0.4, it was analyzed as an independent variable. Finally, we used a non-validated measurement for our main findings on maintaining employees’ mental health status. However, K6 scores increased as subjective mental health status worsened, which may indicate that the measurement was reliable. 

Despite these limitations, this study has several strengths. No previous report has examined general workers’ mental health status in evacuation areas following a nuclear disaster. Also, we clarified factors related to maintaining mental health status, even in harsh workplaces and living environments, following drastic changes due to a disaster. Companies in the evacuation areas ensured employment would contribute to rebuilding communities damaged by the nuclear disaster. Although some companies chose to discontinue business after the disaster, the companies in the present study continued operations to help re-build the community. The companies that made this crucial decision implemented thorough health management for their employees, including measuring radioactivity. Furthermore, our findings show that companies in the evacuation areas introduced methods for encouraging self-care (e.g., regular physical activity, laughing, and having a social network) or employee care by managers, while promoting a well-balanced work and domestic life given the changes in the environment. Consequently, our findings could contribute to employee health management measures in evacuation areas. Also, our results might be applicable for workers’ health management after major disasters in the future.

## 5. Conclusions

Our findings showed drastic changes in the workplace, living environment, and lifestyle of employees in evacuation areas. Despite the harsh environment, the majority of employees in the evacuation area maintained their mental health status, especially those who engaged in regular physical activity, laughed frequently, had a social network, and felt satisfied with their workplace and domestic life. These findings have implications for employee health management measures in evacuation areas to maintain mental health status, even in harsh environments. We hope our work will have implications for future measures addressing workers’ health management after major disasters.

## Figures and Tables

**Table 1:** Basic characteristics of the employees.

Basic Characteristics	Employees in Evacuation Areas		Employees in Non-Evacuation Areas		*p*-Value (χ^2^)
	(*n* = 219)		(*n* = 175)		
	*n* (%)		*n* (%)		
**Age (as of 11 March 2011)**					<0.01 (χ^2^ = 41.4)
Less than 30 years old	50	(22.8)	5	(2.9)	
30–39 years old	36	(16.4)	61	(34.9)	
40–49 years old	75	(34.2)	59	(33.7)	
50 years old or more	58	(26.5)	50	(28.6)	
**Gender**					<0.01 (χ^2^ = 17.7)
Male	181	(82.6)	112	(64.0)	
Female	38	(17.4)	63	(36.0)	
**Occupational category**					0.34 (χ^2^ = 4.49)
Management	28	(12.8)	19	(10.9)	
Clerical work	12	(5.5)	7	(4.0)	
Manufacturing	161	(73.9)	142	(81.1)	
Other	17	(7.8)	7	(4.0)	

*p* < 0.05.

**Table 2:** Differences in risk factors and protective factors between evacuation and non-evacuation areas.

Risk and Protective Factors	Employees in Evacuation Areas		Employees in Non-Evacuation Areas		*p*-Value (χ^2^)
	(*n* = 219)		(*n* = 175)		
	*n* (%)		*n* (%)		
**Stressors on mental health**					
**Change in workplace environment**					
***Amount of overtime work***					<0.01 (χ^2^ = 22.7)
Increase (vs. No change/Decrease)	37	(17.3)	4	(2.3)	
***Work burden***					<0.01 (χ^2^ = 52.2)
Increase (vs. No change/Decrease)	92	(44.0)	18	(10.4)	
***Commute time***					<0.01 (χ^2^ = 191.7)
Increase (vs. No change/Decrease)	163	(76.2)	10	(5.8)	
**Perception of radiation risks**					<0.01 (χ^2^ = 19.4)
Delayed effects High (vs. Low)	103	(48.4)	46	(26.4)	
**Change in living environment**					
***Relocation from an evacuation area***					<0.01 (χ^2^ = 115.9)
Yes (vs. No)	106	(48.4)	0	(0.0)	
***Separation of family members due to the nuclear disaster***					<0.01 (χ^2^ = 104.8)
Yes (vs. No)	138	(63.6)	21	(12.2)	
**Protective factors for mental health**					
***Change in physical activity***					<0.01 (χ^2^ = 22.3)
No change/Increase (vs. Decrease)	145	(66.8)	152	(87.4)	
***Change in sleep time***					<0.01 (χ^2^ = 36.2)
No change/Increase (vs. Decrease)	123	(56.7)	147	(84.5)	
***Social network***					0.56 (χ^2^ = 0.33)
LSNS-6 points ≥ 12 (vs. ≤11)	124	(56.6)	94	(53.7)	
***Frequency of laughing***					0.24 (χ^2^ = 1.36)
Almost every day (vs. 1–5 times per week or less)	57	(27.5)	54	(33.1)	
***Current satisfaction with workplace and domestic life***					<0.01 (χ^2^ = 35.0)
Satisfied with current workplace Yes (vs. No)	82	(37.4)	118	(67.4)	
Satisfied with current domestic life Yes (vs. No)	150	(69.1)	129	(74.6)	0.24 (χ^2^ = 1.40)

*p* < 0.05.

**Table 3:** Mental health status among employees in evacuation areas with changing and current subjective mental health status and Kessler’s 6.

Current Mental Health Status	Employees in Evacuation Areas							
	Change in Subjective Mental Health Status Compared with before the Disaster						K6 Points (SD)	
	Improved		Unchanged		Deteriorated			
Current Subjective Mental Health Status								
Very good	**1**	**(25.0)**	**3**	**(75.0)**	0	(0.0)	0.4	(0.9)
Good	0	(0.0)	**11**	**(100.0)**	0	(0.0)	2.1	(2.6)
Unremarkable	0	(0.0)	**97**	**(78.9)**	26	(21.1)	5.5	(4.3)
Poor	0	(0.0)	6	(8.8)	62	(91.2)	11.4	(4.8)
Very poor	0	(0.0)	0	(0.0)	10	(100.0)	16.4	(5.3)

K6: Kessler-6, SD: Standard Deviation. Bold number: The bolded number indicates employees who maintained their mental health status.

**Table 4:** Distribution of stressors and protective factors for mental health among employees in the evacuation area (maintained vs. deteriorated/unhealthy mental health status).

Age, Gender, Risk and Protective Factors	Maintained		Deteriorated/Unhealthy		*p*-Value (χ^2^)
	(*n* = 112)		(*n* = 104)		
	*n* (%)		*n* (%)		
**Age (as of 11 March 2011)**					
Less than 30 years old	23	(20.5)	27	(26.0)	0.12 (χ^2^ = 5.93)
30–39 years old	13	(11.6)	21	(20.2)	
40–49 years old	46	(41.1)	29	(27.9)	
50 years old or more	30	(26.8)	27	(26.0)	
**Gender**					0.13 (χ^2^ = 2.29)
Male (vs. Female)	97	(86.6)	82	(78.8)	
**Stressors on mental health**					
**Change in workplace environment**					
***Amount of overtime work***					0.15 (χ^2^ = 2.08)
Increase	15	(13.5)	21	(21.0)	
***Work burden***					<0.01 (χ^2^ = 15.1)
Increase	33	(31.1)	58	(58.0)	
***Commute time***					0.39 (χ^2^ = 0.73)
Increase	80	(73.4)	80	(78.4)	
**Perception of radiation risks**					<0.01 (χ^2^ = 12.2)
Delayed effects	41	(36.9)	61	(61.0)	
**Change in living environment**					
***Relocation from an evacuation area***					0.27 (χ^2^ = 1.23)
Yes	58	(51.8)	46	(44.2)	
***Separation of family members due to the nuclear disaster***					0.25 (χ^2^ = 1.30)
Yes	66	(59.5)	69	(67.0)	
**Protective factors for mental health**					
***Change in physical activity***					<0.01 (χ^2^ = 23.0)
No change/Increase	90	(81.8)	53	(51.0)	
***Change in sleep time***					<0.01 (χ^2^ = 16.4)
No change/Increase	78	(69.6)	44	(42.3)	
***Social network***					0.05 (χ^2^ = 3.97)
LSNS-6 ≥ 12 (vs. ≤11)	70	(62.5)	51	(49.0)	
***Frequency of laughing***					0.01 (χ^2^ = 7.08)
Almost every day (vs. 1–5 times per week or less)	38	(35.8)	19	(19.2)	
***Current satisfaction with workplace and domestic life***					<0.01 (χ^2^ = 33.0)
Satisfied with current workplace	63	(56.3)	19	(18.3)	
Satisfied with current domestic life	94	(84.7)	53	(51.5)	<0.01 (χ^2^ = 27.4)

*p* < 0.05.

**Table 5:** Factors related to maintaining employees’ mental health status after the nuclear disaster in the evacuation area.

		Model 1 (Age, Gender, and Stressors on Mental Health)			Model 2 (Added Protective Factors to Model 1)		
		OR (95% CI)		*p*-Value	OR (95% CI)		*p*-Value
**Age (as of 11 March 2011)**		0.98	(0.95–1.01)	0.20	0.96	(0.92–0.99)	0.02
**Gender**	Male	1.97	(0.83–4.68)	0.12	3.55	(1.20–10.5)	0.02
	Female	1.00			1.00		
**Stressors on mental health**							
**Change in workplace environment**							
***Amount of overtime work***	Increase	1.01	(0.83–1.30)	0.77	1.09	(0.84–1.42)	0.53
	No change/Increase	1.00			1.00		0.53
***Work burden***	Increase	0.81	(0.68–0.96)	0.01	0.95	(0.76–1.19)	0.68
	No change/Increase	1.00			1.00		0.68
***Commute time***	Increase	0.90	(0.77–1.14)	0.49	0.98	(0.76–1.26)	0.84
Increase	No change/Increase	1.00			1.00		
**Perception of radiation risks**							
Delayed effects	High	0.81	(0.70–0.94)	0.01	0.87	(0.72–1.05)	0.15
	Low	1.00			1.00		
**Change in living environment**							
***Relocation from an evacuation area***	Yes	1.16	(0.98–1.39)	0.09	1.21	(0.97–1.51)	0.09
	No	1.00			1.00		
***Separation from family members due to the nuclear disaster***							
	Yes	0.73	(0.38–1.38)	0.33	0.46	(0.20–1.04)	0.06
	No	1.00			1.00		
**Protective factors for mental health**							
***Change in physical activity***	No change/Increase				1.31	(1.07–1.62)	0.01
	Decrease				1.00		
***Change in sleep time***	No change/Increase				1.11	(0.91–1.37)	0.30
	Decrease				1.00		
***Social network***	LSNS-6 points ≥12				1.23	(1.01–1.49)	0.04
	LSNS-6 points ≤11				1.00		
***Frequency of laughing***	Almost every day				1.29	(1.02–1.62)	0.03
	1–5 times per week or less				1.00		
***Satisfaction with current workplace and domestic life***							
Satisfied with current workplace	Yes				1.38	(1.12–1.69)	<0.01
	No				1.00		
Satisfied with current domestic life	Yes				1.26	(1.01–1.57)	0.04
	No				1.00		

OR: Odds Ratio, CI: Confidence Interval, *p* < 0.05.
